# The changing nature of patient attributes available for matching.

**DOI:** 10.23889/ijpds.v7i3.2079

**Published:** 2022-08-25

**Authors:** Yu Deng, Lacey Gleason, Adam Culbertson, Don Asmonga, Shaun Grannis, Abel Kho

**Affiliations:** 1 Northwestern University; 2 The Pew Charitable Trusts; 3 Regenstrief Institute

## Objectives

Patient matching rates between organizations can be as low as fifty percent. Challenges to matching include the variation in quality and availability of patient attributes. Here we describe the changing nature of patient attributes available over the past 11-years across a diversity of care settings in the United States.

## Approach

Our expert panel identified 64 patient attributes that are currently used or could potentially be candidates for patient matching. We identified a national sample of 14 health care sites who sent us aggregated information on the 64 patient attributes from 2010 to 2020 (inclusive). The information included overall counts and percent availability, overall counts and percent availability by race, and counts and availability by year. Only patients having at least one visit to the site since 2010 and who were between 18 and 89 years of age at time of extraction were included.

## Results

The aggregated results revealed that first name, last name, gender, postal codes, and date of birth are highly available (>90%) across healthcare organizations and time. Patient reported social security number, work phone number, and emergency contact declined markedly, potentially reflecting privacy concerns. Email addresses (from 18.0% to 63.7%) and phone numbers (from 14.7% to 69.4%) increased greatly over the past 11 years. Novel patient matching attributes such as blood type, facial image, thumb print, or eye color are rarely collected across sites for all years. We observed emerging attributes including sexuality, occupation, and nickname with a small number of sites collecting these over 70%, reflecting the feasibility of wider adoption in the future.

## Conclusion

In this study, we examined the availability of 64 patient attributes across 14 sites from 2010 and 2020. Our findings could inform policy makers and readers about patient attributes that are used for current patient matching and emerging data attributes that could be considered for incorporation into future matching algorithms.

